# Supplementary material for: Uncovering low-frequency vibrations in surface-enhanced Raman of organic molecules
Source: Nat Commun. 2024 Aug 7;15:6733. doi: 10.1038/s41467-024-50823-x (PMC11306350; doi:10.1038/s41467-024-50823-x)
Supplement: Supplementary file 1 — Supplementary Information [file 41467_2024_50823_MOESM1_ESM.pdf]

Supplementary Information

Uncovering low-frequency vibrations in surface-enhanced Raman of organic molecules

Alexandra Boehmke Amoruso[1], Roberto A Boto[2,3], Eoin Elliot[1], Bart de Nijs[1], Ruben Esteban[2,3],Tamás Földes[4], Fernando Aguilar-Galindo[3,5,6], Edina Rosta[4], Javier Aizpurua[3,7,8*], Jeremy J Baumberg[1*]

[1] NanoPhotonics Centre, Cavendish Laboratory, J J Thomson Avenue, University of Cambridge, CB3 0HE, UK

[2] Centro de Física de Materiales CFM-MPC (CSIC UPV/EHU), Paseo Manuel de Lardizabal 5, 20018 Donostia-San Sebastián, Spain

[3] Donostia International Physics Center (DIPC), Paseo Manuel de Lardizabal 4, 20018 Donostia-San Sebastián, Spain

[4] Department of Physics and Astronomy, University College London, London WC1E 6BT, UK

[5] Institute for Advanced Research in Chemical Sciences (IAdCHEM), Universidad Autónoma de Madrid, 28049 Madrid, Spain

[6] Departamento de Química, Universidad Autónoma de Madrid, 28049 Madrid, Spain

[7]Ikerbasque, Basque Foundation for Science, Bilbao, Spain

[8]Dpt. of Electricity and Electronics, University of the Basque Country, UPV/EHU, 48940 Leioa, Spain

[*] corresponding author email: jjb12@cam.ac.uk, aizpurua@ehu.eus

Supplementary Information Contents

I. Supplementary Note 1 | Analytes
- Table S1 | List of molecule names and their *CAS* identifiers
- Figure S1 | Comparison of Raman of bulk crystal and SAM

II. Supplementary Note 2 | Fitting bosonic background to THz SERS
- Figure S2 | Fitting equation (1) to measured THz SERS
- Figure S3 | Fit parameter T

III. Supplementary Note 3 | Molecular identification by low-frequency spectrum
- Figure S4 | Thirty most representative spectra for each molecule

IV. Supplementary Note 4 | Exponential background fit
- Figure S5 | Exponential fit to low-frequency region of spectra
- Figure S6 | Low-frequency exponential decay rates

V. Supplementary Note 5 | Sample characterization
- Figure S7 | NPoM sample characterization with darkfield spectroscopy
- Table S2 | Comparison of measured and simulated characterization of NPoM gap thickness
- Figure S8 | Diagram of molecular length, height, and tilt angle

VI. Supplementary Note 6 | Width of experimental molecular Raman lines
- Figure S9 | Molecular Raman peak linewidths of representative spectra

VII. Supplementary Note 7 | Density Functional Theory simulation in different molecular configurations
- Figure S10 | Illustrations of different Au structures compared in Fig. S11
- Figure S11 | Influence of Au on simulated Raman spectra
- Figure S12 | Influence of gap thickness on simulated Raman spectra

VIII. Supplementary Note 8 | Animations of the collective vibrational modes

IX. Supplementary Note 9 | THz single-molecule vibrational modes

Supplementary Note 1 | Analytes

Molecules (**1**) – (**7**) (Table S1) are formed into SAMs on template-stripped Au mirrors for the experiments here.

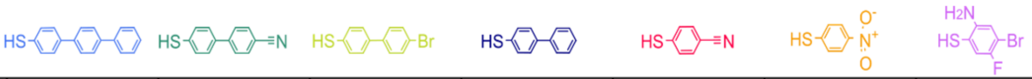

	(1)	(2)	(3)	(4)	(5)	(6)	(7)
**Molecule name**	terphenyl-4-thiol	4′-mercaptobiphenyl-carbonitrile	4′-bromo-4-mercapto-biphenyl	biphenyl-4-thiol (BPT)	4-thiobenzo-nitrile (MBN)	4-nitrothio-phenol	2-amino-4-bromo-5-fluorobenzenethiol
**CAS**	90589-98-3	64409-12-7	220805-21-0	19813-90-2	36801-01-1	1849-36-1	1474100-85-0

Table S1 | List of molecule names and *Chemical Abstracts Service (CAS)* identifiers.

For comparison, the THz Raman spectra of these molecules in bulk crystal phase were also measured (Figure S1). The molecules were purchased in powder-crystal form and measured on an aluminium substrate with the same THz Raman microscope setup as described in Methods.

The measured Raman of the powdered crystal (dark) and SAM (light) phases of molecules (**1**,**4**) are normalized for comparison in the THz region. In the high-frequency region (Fig. S1b,d), shifts in peak positions are observed between the two phases of each molecule. In the low-frequency region (a,c),the spectra of the powders have distinct and slightly narrower peaks compared to those of the SAM. This gives additional evidence that broadening of the low-frequency peaks occurs due to heterogeneity in the SAM.

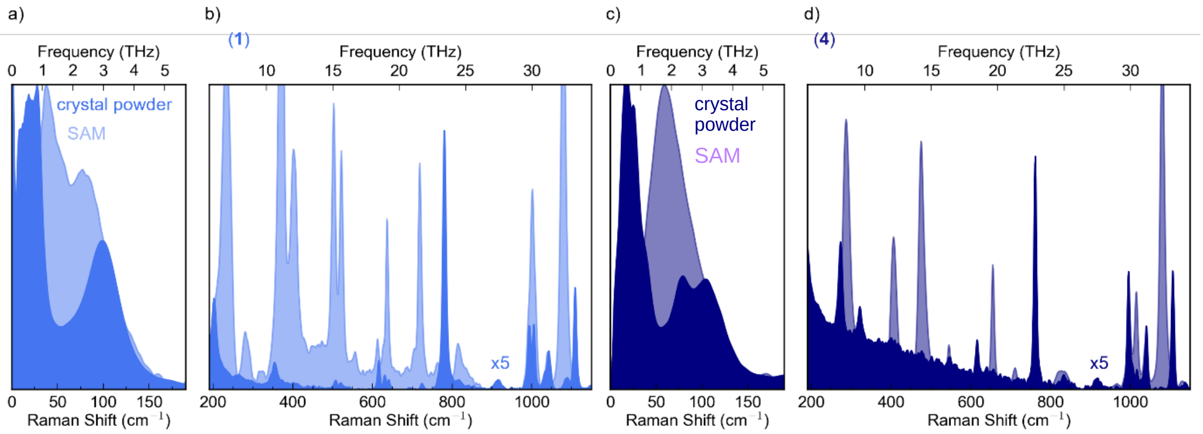

Figure S1 | Comparison of Raman of the bulk crystals and molecular-monolayer of (a,b) molecule (**1**) and (c,d) molecule (**4**). The high-frequency regions of the powder spectra are scaled up x5 for comparison of peak positions.

Supplementary Note 2 | Fitting bosonic background to THz SERS

The measured SERS (black dashed lines) of molecules (**1**,**4**,**7**) are compared with the best-fit electronic Raman scattering model (Eq.(1) in main text) [Fig. S2a]. Initially, the peaks are approximately removed with a simple algorithm to produce a first approximation of the background (blue). The model is then fit to this curve using a simulated annealing algorithm that infinitely penalizes results that produce a fit-subtracted spectrum with nonphysical negative intensities.

Figure S2(a) shows the optimal fit (red) which is mainly matching the higher wavenumber components, as well as the broad component which models the EF factor (yellow). This EF factor is modelled as a broad single or double gaussian and constrained to the position(s) of the plasmon resonance(s) of the particular NPoM, separately measured with darkfield spectroscopy. Gaussians provide a better fit than Lorentzian lines. The FWHM of the gaussians are constrained to 700-1500 cm^{-1} (50-100nm), matching the DF resonances. In some cases, the near-field EF enhancement within an NPoM appears to be different from the resonances measured in the far-field scattering spectrum, as also seen in previous work [DOI 10.1021/acsphotonics.5b00707].

One fitting parameter (A) quantifies the strength of the electronic Raman susceptibility of Au, χ. At high Stokes Raman Shifts, A gives the height of the background. The temperature parameter T (Eq.(1) in main text) is constrained to acceptable values (288-340 K), and matches the antiStokes side well. The best-fit values of parameter T from the ERS fits of each molecule (Fig. S2), fall within the constraints giving values close to room temperature (293 K) (Fig. S3).

The fit-subtracted spectrum (expt – fit) for each molecule (Fig. S2b, red) is plotted on a log scale to emphasize how close the higher wavenumber backgrounds are now to zero. Upon inspection, it is clear that the ERS model cannot account for the remaining steep background below 200 cm^{-1}, no matter what improvements are made to the fitting algorithm (see Fig. S4). Because the source of the remaining background is unknown, we chose to classify the spectra before removing it.

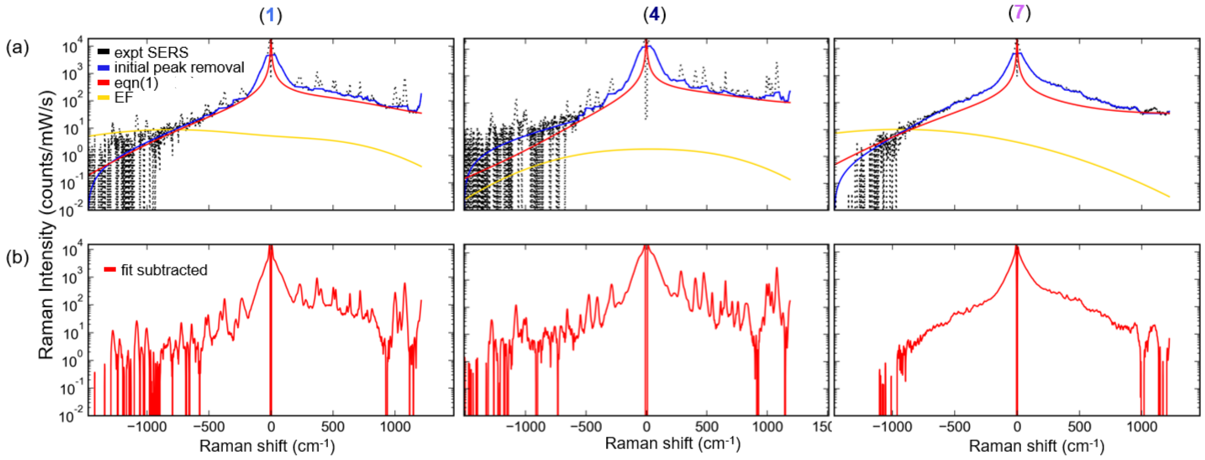

Figure S2 | Exemplars of fitting equation (1) of main text, to a few measured THz SERS of molecules (**1**,**4**,**7**). (a) Measured data (black dashed), initial extracted background (blue), EF plasmonic enhancement (yellow), and final fit (red). (b) Data with this background fit now subtracted, showing remaining SERS peaks and background that is above the bosonic floor.

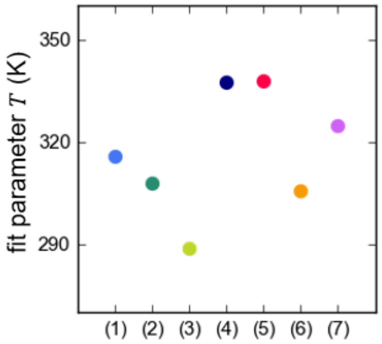

Figure S3 | Extracted average fit parameter T of molecules (**1**-**7**).

Supplementary Note 3 | Molecular identification by low-frequency spectrum

After subtracting the bosonic background fit (Fig. S2b), the thirty most typical spectra of each molecule were used to classify the low-frequency spectra (<200cm^{-1}), extracted in the following manner.

The full dataset for each molecule consists of a series of more than 10 spectra measured successively from each of >100 single NPoMs. Initially, the background fit (Note 2) was subtracted from each spectrum. Then, an algorithm was applied to the series of spectra for each NPoM to separate transient spectra from the stable spectrum, referred to as the "nanocavity spectrum." The average nanocavity THz spectrum for each NPoM is then restricted to the region 25-200 cm^{-1} and each normalized to one (Scikit-learn Preprocessing normalize, norm = 'l1') in order to compare the shape of the low-frequency regions. Spectra with low signal-to-noise ratios were dropped and the spectra aligned on the same x-axis scale. The thirty most representative nanocavity spectra of each molecule (Fig. S4) are identified by calculating the Mahalanobis distance of each from the centroid of the dataset for each molecule and using the thirty with the shortest distances.

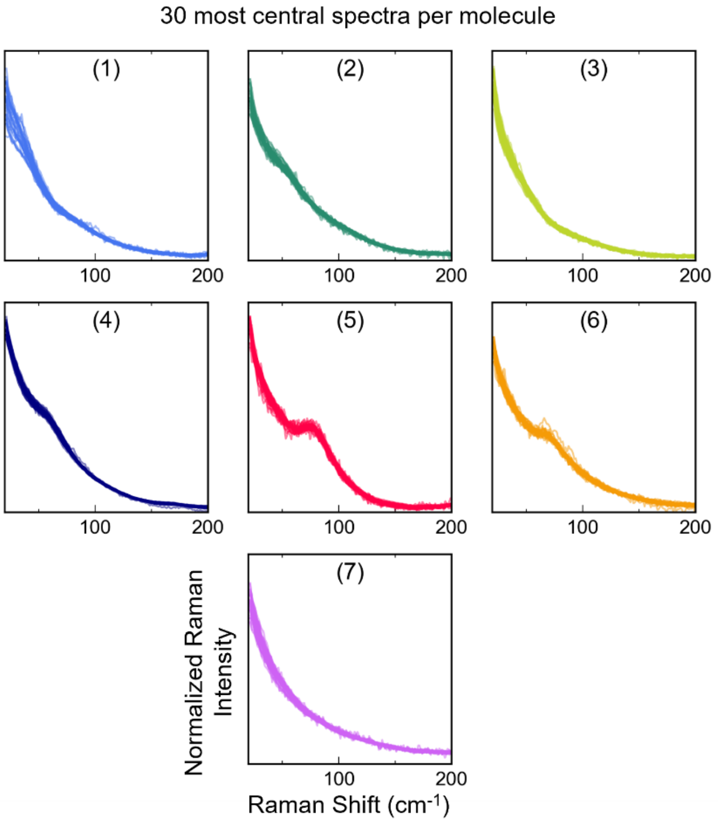

Figure S4 | Thirty most representative spectra for each molecule, showing their strong overlap and repeatability.

A variational Bayesian Gaussian Mixture model implemented with Scikit-learn was used to cluster the set of these thirty spectra of each of the seven molecules (tied covariance type, K-Means initialization) (Fig. 2b).[1] The model, covariance type, and number of components were selected by maximizing the normalized mutual information score. A score of 0.99 was achieved, showing that the shape of the low-frequency spectrum correlates strongly with molecular structure.

Supplementary Note 4 | Exponential background fit

An additional contribution with exponential shape in the low-frequency region of the SERS spectra was observed after removing the bosonic background (Eqn.(1) of main text). The fitting of this contribution for molecules (**1**) and (**7**) is shown in Fig. S5. First, the minima between peaks in the low-

frequency spectrum are found and used to define an initial exponential fit. This is used as an initial guess (red line, left Fig. S5a,c) to extract the optimised parameters using a simulated annealing algorithm. Again this infinitely penalizes fits giving negative intensities in the residuals (blue line, left Fig. S5b,d). The final fit (red line, right Fig. S5a,c) results in a fit-subtracted residual spectrum which preserves the THz molecular peaks (blue line, right Fig. S5b,d).

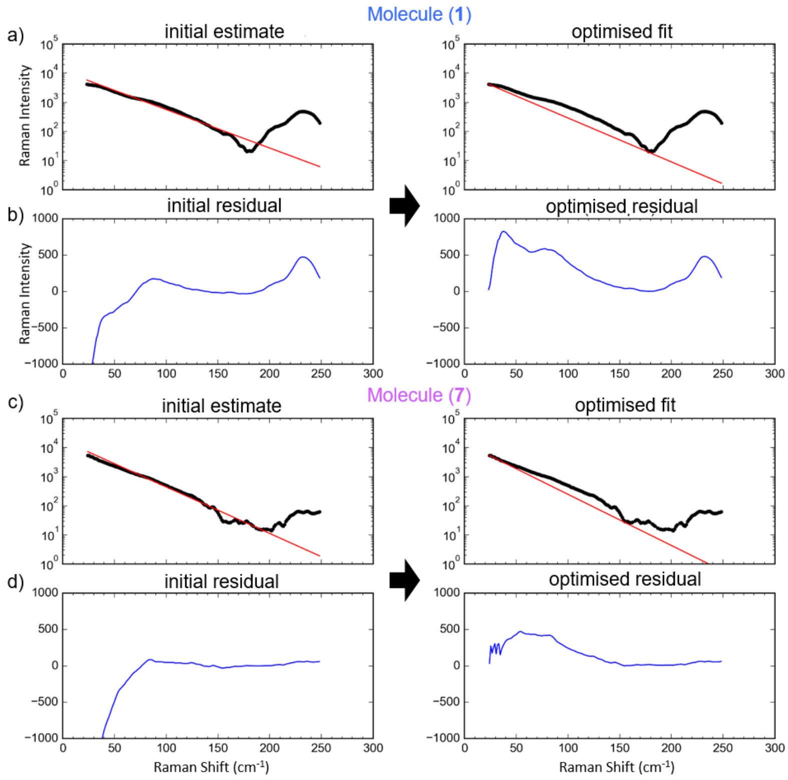

Figure S5 | Exponential fit (red) to low-frequency region (25-200cm^{-1}) of THz SERS spectra of (a,b) molecule (**1**) and (c,d) molecule (**7**). Left side shows initial estimate for fit, while right side shows optimised exponential fit. Black line shows data.

The decay rates given by the above exponential fits for each of the seven molecules are ~30 ± 5 cm^{-1} (Fig. S6). Even molecule (**7**), whose SERS spectrum does not contain stable molecular Raman peaks ,gives the same fit value. This suggests that this exponential factor in the SERS spectrum is molecule independent and arises from the Au nanostructure. The molecular-dependent spectra of each SAM,resulting from subtracting the ERS and exponential backgrounds, are shown in Figure 3 of the main text.

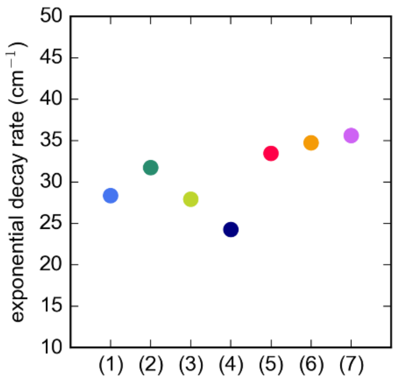

Figure S6 | Low-frequency exponential decay rates for Au NPoMs containing molecules (**1**-**7**).

Supplementary Note 5 | Sample characterization

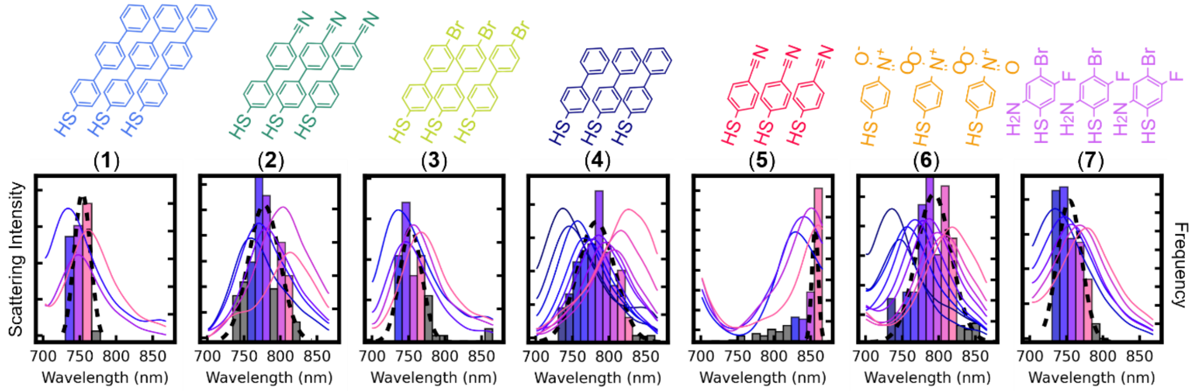

Figure S7 | NPoM sample characterization using darkfield spectroscopy for molecules (**1**) – (**7**). Histogram shows the number of NPoMs with each DF peak wavelength, coloured lines show corresponding average spectra for each histogram bin.

The darkfield (DF) scattering spectra of each individual NPoM are measured and the long-wavelength coupled plasmon peak wavelength is analysed in a histogram for each molecule (Fig. S7). A gaussian function is fit to these distributions, and their centre wavelengths are extracted (Table S2).

The centre wavelength is used to calculate the gap size set by the SAMs of each molecule using a NPoM quasi-normal mode tool (also published online[2]) to compare with expected gap thickness (Table S2). We assume the observed resonance is the (10) coupled mode of the 80-nm Au nanoparticle with a 12-nm typical circular facet radius[3] containing a SAM with index of refraction n=1.45.[4] Using these assumptions gives gap thicknesses in reasonable agreement with prior work for molecule (**4**) (from phase-modulated ellipsometry thickness of 1.3 ± 0.1 nm)[5] and with simulations. The agreement with simulation is not as good for the shorter molecules (**6**) and (**7**), but is improved for the latter by using instead n=1.6.[5] In the case of molecule (**7**), which did not form a consistent SAM, the (10) mode seems to be red-shifted out of view into the infrared and the (20) peak is instead observed in this spectral
range. The (20) resonance of a 12nm circularly faceted 80nm NPoM agrees better with the gap thicknesses from DFT for molecule (**7**), if using n=1.77, similar to the literature value[6].

These DF gaps are then used as the thickness in Fig. 3d. They are compared to the maximum extended length of each molecule according to DFT (an approximation calculated by adding together the interatomic distances along the backbone of the molecule from the Au to the tailgroup), as well as to the optimal gap thicknesses used in the DFT simulations shown in Fig. 3a,b (Table S2). To select the thickness used in DFT, SERS spectra of single molecules between two parallel planes of Au atoms with varying gap thicknesses were simulated with DFT (Fig. S12), and the spectrum best matching experiment selected (Fig. 3a,b).

**molecule**	**(1)**	**(2)**	**(3)**	**(4)**	**(5)**	**(6)**	**(7)**
**DF peak (nm)**	753	778	753	782	856	793	754
**DF gap thickness (Å)**	19	15	19	15	9	12	9
**optimal DFT gap (Å)**	12	12	14	10	8	8	8

**extended length (Å)**	14.8	12.0	11.5	10.7	8.1	7.9	7.5

Table S2 | Comparison of measured and simulated characterization of NPoM gap thickness. The DF gap thickness is calculated from the DF peak position using the quasi-normal mode tool referenced in Note 5. The optimal DFT gap is selected by comparing the DFT simulations of the SERS spectrum of each molecule in a range of gap sizes, to the measured SERS spectrum. The extended length is approximated by summing together the bond lengths along the backbone of each of the simulated equilibrium molecular structures.

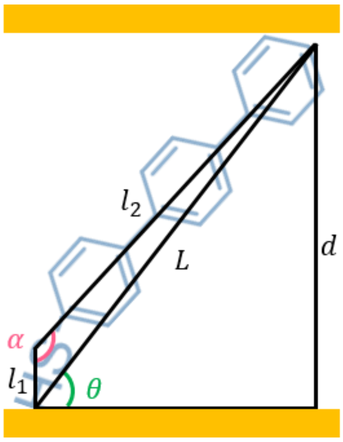

Figure S8 | Diagram of molecular length, height, and tilt angle 90-θ for molecule (**3**).

The gap thickness can be used to estimate the tilt angle from DFT-simulated values of the molecular Au-S bond length l_1, the molecular 'backbone' length l_2 taken from DFT, and the Au-S-Ph angle α~100° from DFT. With d the height of the terminal atom, L can be calculated to give effective tilt

$$\sin(\theta) = d/L, \quad L^2 = l_1^2 + l_2^2 - 2l_1 l_2 cos(\alpha) \tag{S1}$$

For example, from the simulated structure of (**1**) in a 14Å gap, $l_1 = 2.5$Å, $l_2 = 14.1$Å, $\alpha = 99.8°$, so according to Eq.(S1), L=14.7Å. For the DFT gap thickness $d = 12$ Å, the tilt angle 90°−θ=32°, which is indeed a realistic tilt angle. In this case, for the measured DF gap thickness of 19Å to be correct, there would be 5Å above the terminal atom to the top Au surface. More realistic DFT simulations will need to take into account the intermolecular interactions of the molecules in a SAM on the Au surface to give optimised tilt angles.

Supplementary Note 6 | Width of experimental molecular Raman lines

The full-width-at-half-max (FWHM) of the Raman peaks of the representative measured spectrum of each molecule are found by fitting a Lorentzian function to each peak (results are similar if a Gaussian function is used instead). The FWHM increases threefold for vibrational modes below 200 cm^{-1}, except in the spectra of picocavities (magenta stars).

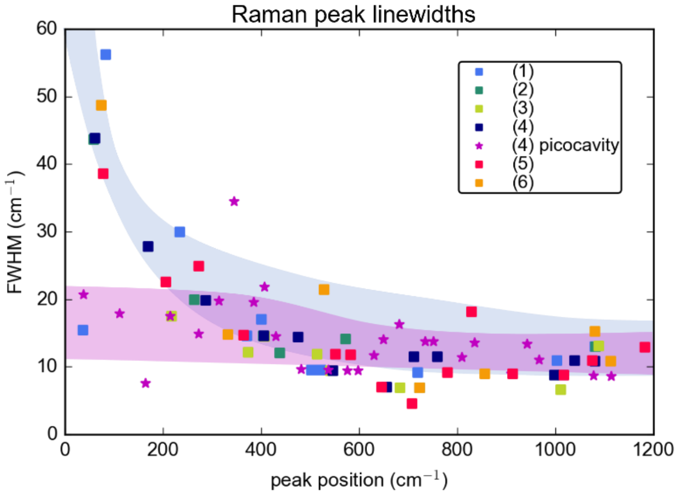

Figure S9 | Molecular SERS peak linewidths of representative spectra of molecules (**1**) – (**6**), compared to those of a picocavity spectrum of molecule (**4**) (stars).

Supplementary Note 7 | Density Functional Theory Simulation in different molecular configurations

The DFT simulations in the main text consider a molecule placed between two metal planes (Fig. S10d). Here, we also consider other possible metal-molecule configurations.

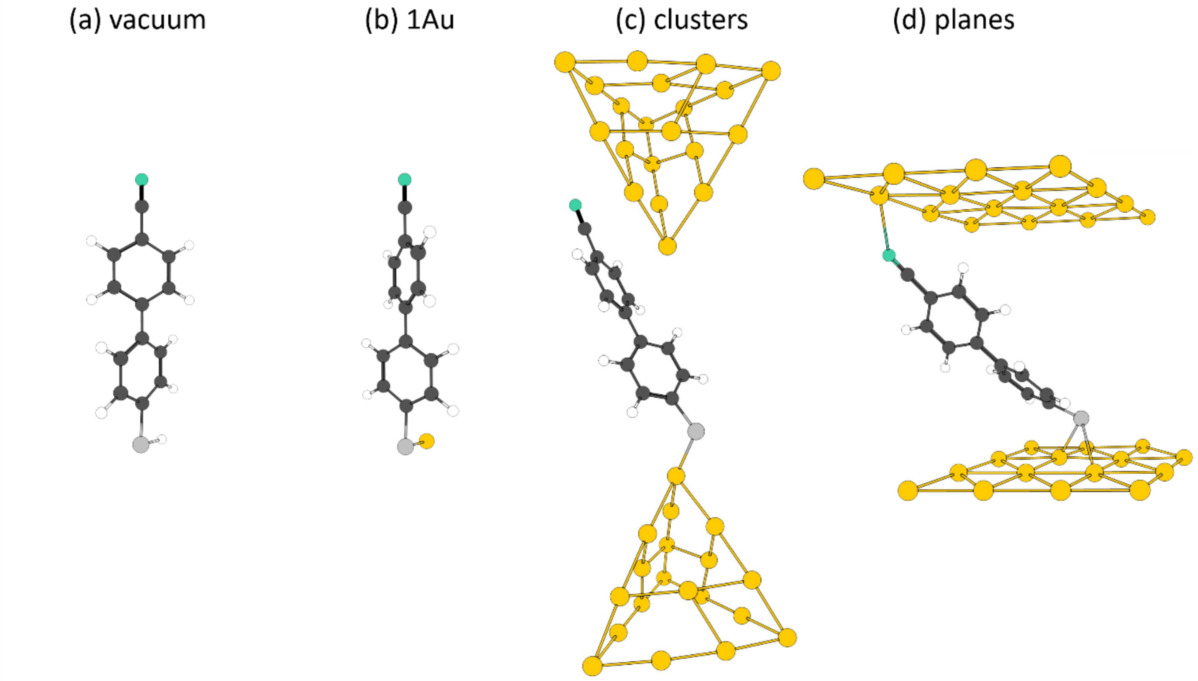

Figure S10 | Configuration of Au atomic blocks and molecule (**2**) for (a) vacuum (b) single Au atom, (c) clusters, and (d) parallel planes.

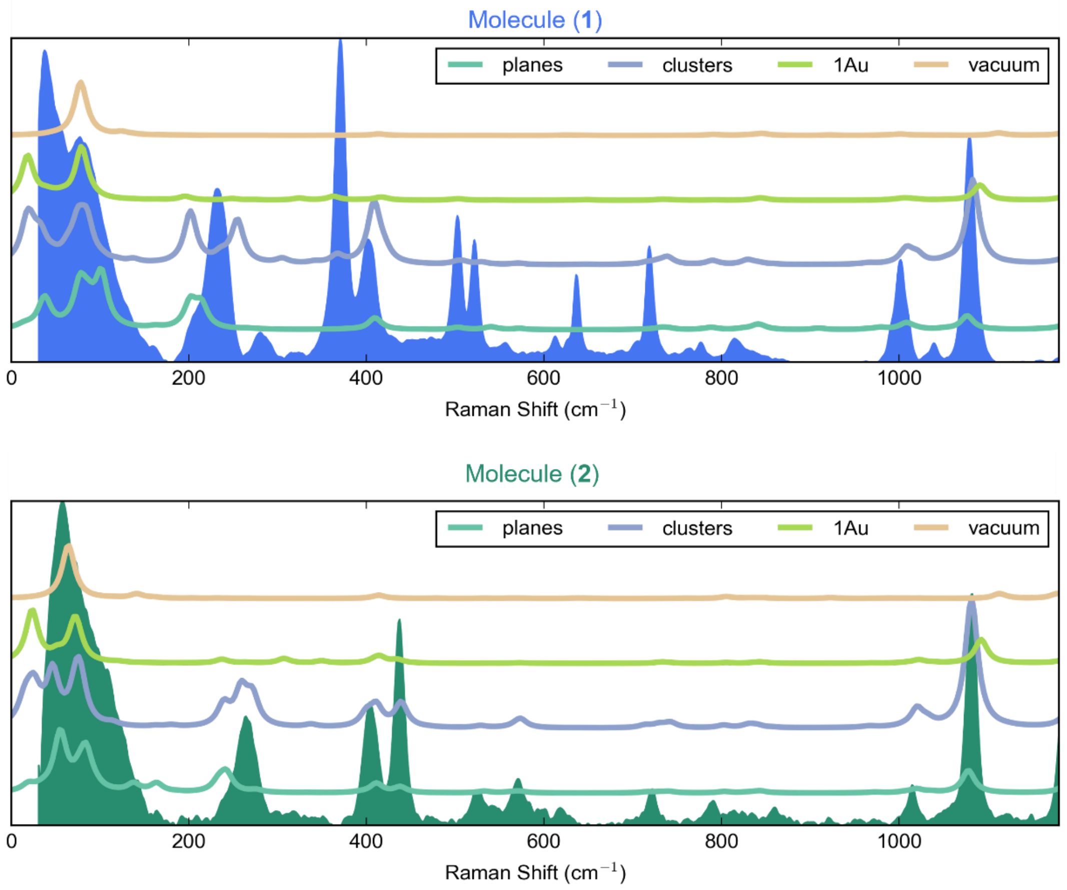

Figure S11 | Influence of Au on simulated Raman spectra of molecules (**1**,**2**). The DFT-simulated Lorentzian-broadened Raman intensities (omitting any modes <25cm^{-1}) are overlaid over the measured SERS spectrum (shaded).

The measured SERS of the SAMs of molecules (**1**) and (**2**) in NPoMs (shaded spectra in Fig. S11, data as in Fig. 3a,b) are compared to normalized DFT-simulated Raman spectra of a single molecule bound by its thiol to different Au structures (spectra shown in Fig. S11). These include monolayer planes of Au atoms, compact tetrahedral clusters of Au atoms with the molecule bound to one point, and single Au atoms, as well as no Au present (vacuum) (Fig. S10). These different simulations demonstrate the impact of the Au nanostructure on the molecular Raman spectrum (beyond the plasmonic enhancement factor EF). In particular, the low-frequency spectra change drastically. In order to correctly model the THz modes, it is clear that a high level of metal detail is required since the Au atoms form a macro-system coupled to the single molecule (and others surrounding it). However, we find that parallel planes of Au atoms simulating the top and bottom facets of the NPoM are sufficient to reproduce qualitatively the relative enhancement of many of the THz modes, and thus they improve the agreement with the SERS measurement, as compared to atomistic models that include one gold atom bound to the molecule or no gold atoms (vacuum). To further improve the agreement between the simulated and the measured Raman spectra it is necessary to include also the intermolecular effects in the SAM, by simulating a set of molecules between the Au planes. We present progress towards this challenging endeavour for molecules **4** and **5** in the main text.

Molecules (**1**-**7**) were also simulated in several different gap sizes between the plane Au facets. The simulated Raman spectra most similar to the measured spectra are shown in Fig. 3. In Fig. S12 the measured spectra of molecules (**1**,**2**) are shown shaded, with spectra showing the normalized DFT-simulated Raman when varying the distance between the two parallel planes of Au atoms, from 8 to 14 Å (bottom to top). A single molecule was bound to one plane by its thiol end, and was allowed to

relax as the distance of the second plane was changed. Because the orientation of the molecule was allowed to change compared to the optical E field polarized normal to the planes, the Raman peak intensities across the spectrum vary considerably. Additional effects of charge redistribution related to tilt angle also cause shifts in peak positions. In order to fix the tilt angle, future work is required to simulate larger arrays of molecules in the nanogap.

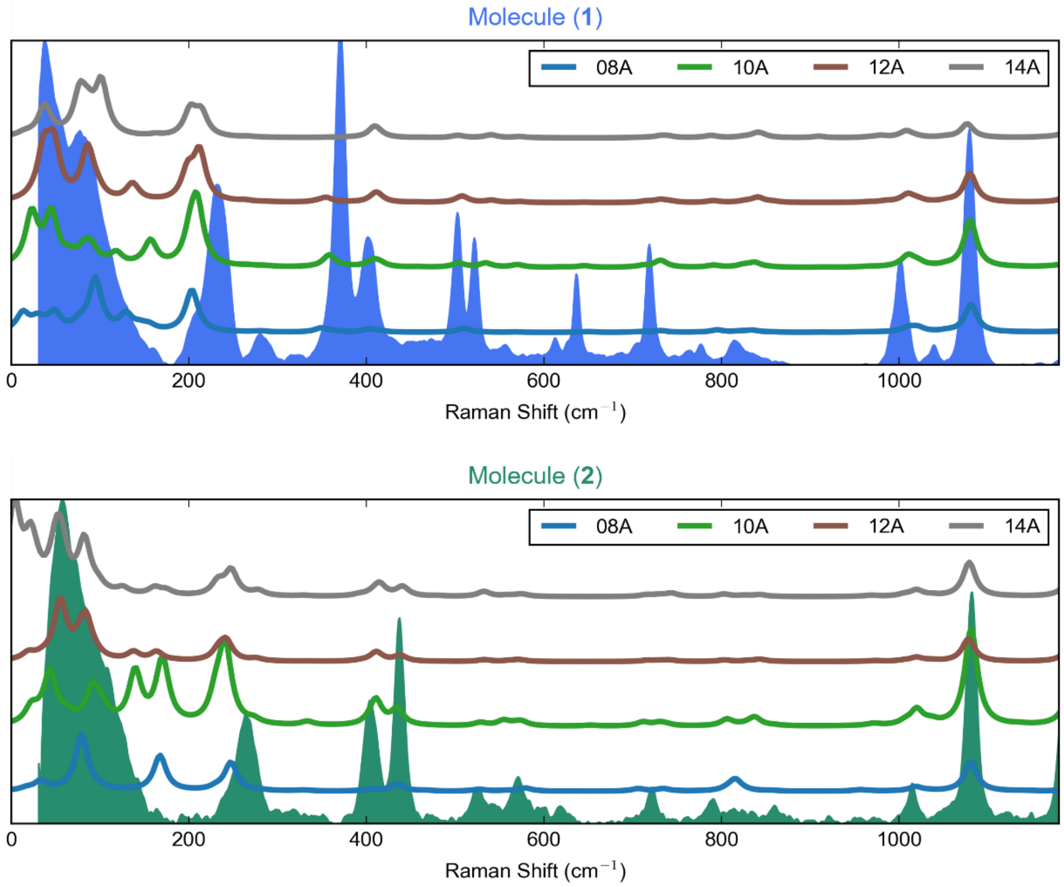

Figure S12 | Influence of gap thickness on simulated Raman spectra of molecules (**1**,**2**). The DFT-simulated Lorentzian-broadened Raman intensities (omitting any modes <25cm^{-1}) are overlaid over the measured SERS spectrum (shaded).

Supplementary Note 8 | Animations of the collective vibrational modes

In the Supporting Material we provide animations of the atomic motions of the strongest vibrational modes in the 25-200 cm^{-1} range for the model SAMs of three BPT (molecule **4**) and four MBN (molecule **5**) molecules, respectively, located between two parallel gold monolayers. We also include animations of the corresponding vibrational modes of a single BPT and MBN molecule. The atoms of hydrogen, carbon, nitrogen, sulfur and gold are depicted in the animation as white, grey, blue, brown and yellow spheres, respectively. The thin blue arrows in the animation point towards the direction of the atomic displacements.

Relevant information about these animations is contained in Table S3 (molecule **4**) and Table S4 (molecule **5**). The three left-most columns correspond to the model SAMs, and the other three to the single molecule vibrations. We include the filename of the animations (columns 1,4), the wavenumber of the selected vibrational modes in cm^{-1} (columns 2,5), and the description of the vibrational modes (columns 3,6) based on the following nomenclature: 1) shearing modes when the atoms of the molecule move mostly in the plane of the gold layers; 2) tapping modes when the atomic

displacements are nearly out of plane of the gold layers; 3) twist modes when the vibration consists primarily of a frustrated rotation; 4) inter-ring bending modes (only for molecule **4**) when the relative motions of the two phenyl rings are in antiphase. We note that these descriptions are simplified as the vibrational patterns can involve multiple types of motion.

<table>
<tr><th colspan="3">Collective vibrational modes</th><th colspan="3">Single-molecule vibrational modes</th></tr>
<tr><th>Filename</th><th>Wavenumber (cm^{-1})</th><th>Description</th><th>Filename</th><th>Wavenumber (cm^{-1})</th><th>Description</th></tr>
<tr><td>SAM_BPT_mode7.gif</td><td>36</td><td>Tapping (localised in one molecule)</td><td>1mol_BPT_mode4_.gif</td><td>47</td><td>Tapping</td></tr>
<tr><td>SAM_BPT_mode14.gif</td><td>66</td><td>Shearing (localised in one molecule)</td><td>1mol_BPT_mode7.gif</td><td>72</td><td>Shearing</td></tr>
<tr><td>SAM_BPT_mode23.gif</td><td>136</td><td>Inter-ring bending (localised in two molecules)</td><td rowspan="2">1mol_BPT_mode8.gif</td><td rowspan="2">141</td><td rowspan="2">Inter-ring bending</td></tr>
<tr><td>SAM_BPT_mode24.gif</td><td>143</td><td>Inter-ring bending (localised in one molecule)</td></tr>
<tr><td rowspan="2">SAM_BPT_mode26.gif</td><td rowspan="2">178</td><td rowspan="2">Shearing+sulfur displacement</td><td>1mol_BPT_mode9.gif</td><td>166</td><td rowspan="2">Shearing+sulfur displacement</td></tr>
<tr><td>1mol_BPT_mode10.gif</td><td>186</td></tr>
</table>

Table S3 | Guide to the animations showing the atomic displacements corresponding to collective vibrational modes of the model SAM consisting of three BPT molecules (molecule **4**, columns 1-3) and of a single BPT molecule (columns 4-6). In both cases the molecules are located between two gold layers. **Columns 1,4**: Filenames of animation showing the atomic motions of the vibrational modes. **Column 2,5:** Calculated wavenumbers of the vibrational modes, scaled by a factor 0.992 (column 2) and 0.985 (column 5). **Columns 3,6**: Descriptions of the vibrational modes. Note, the scaling factors are different here because of treating the case of the single and multiple molecules within periodic boundary conditions, as compared to the single molecule-case with no periodic boundaries of main text Fig. 3 (with scaling factor0.986).

Collective vibrational modes			Single-molecule vibrational modes		
Filename	Wavenumber (cm^{-1})	Description	Filename	Wavenumber (cm^{-1})	Description
SAM_MBN_mode8.gif	45	twist	1mol_MBN_mode3.gif	33	twist
SAM_MBNl_mode9.gif	57	shearing /tapping	1mol_MBN_mode5.gif	77	shearing /tapping
SAM_MBN_mode10.gif	63				
SAM_MBN_mode11.gif	66				
SAM_MBN_mode12.gif	67				
SAM_MBN_mode13.gif	72				
SAM_MBN_mode14.gif	76				
SAM_MBN__mode27.gif	180	C-N shearing	1mol_MBN_mode8.gif	188	C-N shearing
SAM_MBN_mode28.gif	181				
SAM_MBN_mode29.gif	182				
SAM_MBN_mode30.gif	190				
SAM_MBN_mode31.gif	194				
SAM_MBN_mode3.gif	194				

Table S4 | Guide to the animations showing the atomic displacements corresponding to collective vibrational modes of the model SAM consisting of four MBN molecules (molecule **5**, columns 1-3) and of a single MBN molecule (columns 4-6). In both cases the molecules are located between two gold layers. **Columns 1,4**: Filenames of animations showing the atomic motions of the vibrational modes. **Column 2,5:** Calculated wavenumbers of the vibrational modes, scaled by a factor 0.993 (column 2) and 0.992 (column 5). **Columns 3,6**: Descriptions of the vibrational modes. Note, the scaling factors are different here because of treating the case of the single and multiple molecules within periodic boundary conditions, as compared to the single molecule-case with no periodic boundaries of main text Fig. 3 (with scaling factor 0.986).

Supplementary Note 9 | THz single-molecule vibrational modes

We illustrate in Table S5 the characterisation of the two most intense vibrational modes in the 25-200 cm^{-1} range for each single molecule located between two monolayer planes of fifteen and sixteen atoms of gold.

Molecule	Raman Shift (cm^{-1})	Illustration	Description of tail motion
1 (TPT)<br>Optimal DFT gap: 12 Å	48	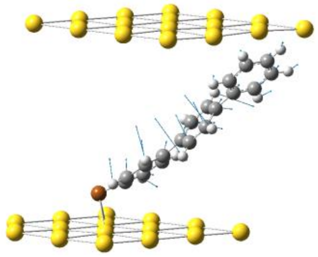	Tapping
	86	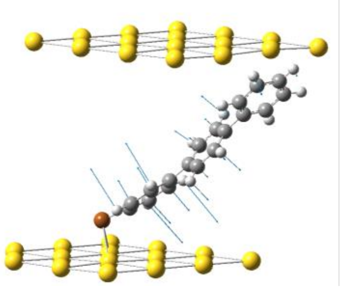	Ring twist
2 (NC-BPT)<br>Optimal DFT gap: 12 Å	56	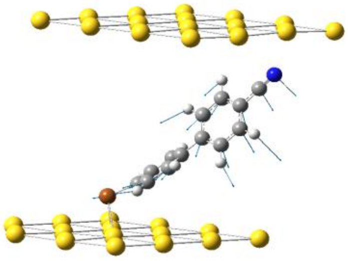	Shearing + tapping
	84	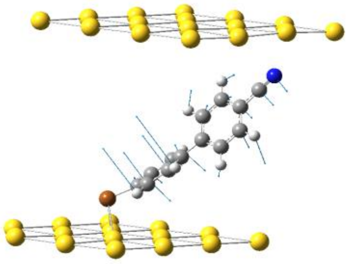	Ring twist + shearing

3 (Br-BPT)<br>Optimal DFT gap: 14 Å	47	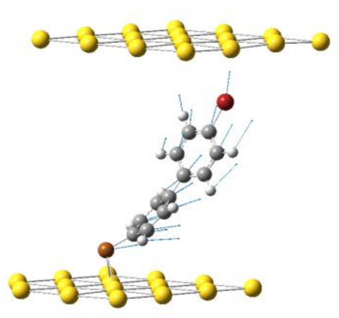	Tapping
	82	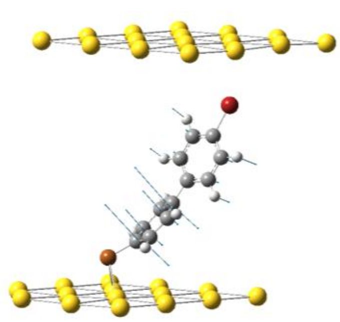	Ring twist
4 (BPT)<br>Optimal DFT gap: 10 Å	44	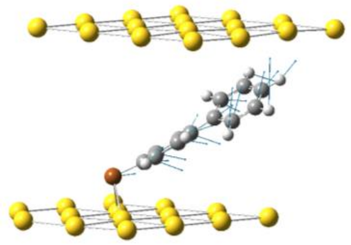	Tapping + ring twist
	137	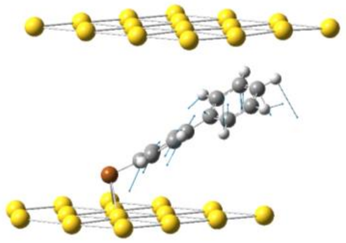	Shearing + tapping

5 (MBN)<br>Optimal DFT gap: 8 Å	76	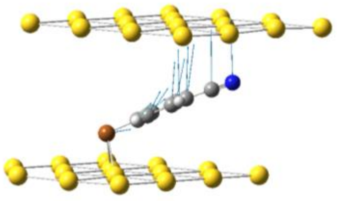	Tapping
	177	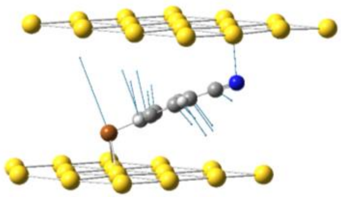	Tapping
6 (NPT)<br>Optimal DFT gap: 8 Å	68	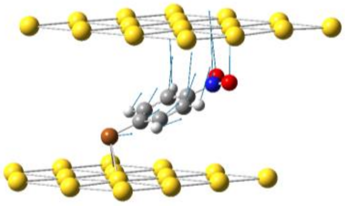	Tapping
	110	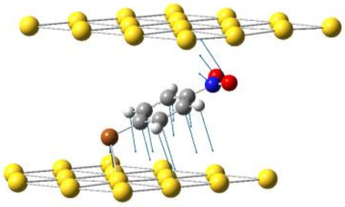	Tapping

**7 (ABT-Br-F)**<br>**Optimal DFT gap: 8 Å**	39	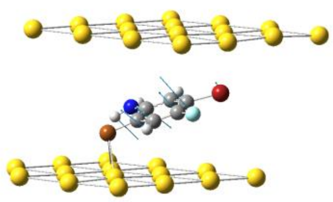	Shearing
	132	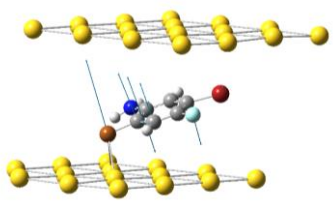	Tapping

Table S5 | Characterization of the two most intense Raman vibrational modes in the THz region (25-200 cm^{-1}; 0.7-6 THz) for each of the seven selected molecules between two gold layers. **Column 1:** Abbreviation of the name of the molecules along with the numerical code that we use in the manuscript to identify the molecules (see Table S1). We also include the optimal gap thickness used in the DFT simulations (see Table S2 for further details). **Column 2**: Calculated wavenumbers of the vibrational modes scaled by a factor 0.986. **Column 3:** Illustrations of the atomic motions of the strongest vibrational modes in the 25-200 cm^{-1} range. The atoms of hydrogen, carbon, nitrogen, oxygen, fluorine, sulfur, bromine and gold are depicted as white, grey, dark blue, red, light blue, brown, magenta and yellow spheres, respectively. The thin blue arrows point towards the direction of the atomic displacements. **Column 4:** Description of the vibrational modes using the nomenclature introduced in Supplementary Note 8. We note that these descriptions are simplified as the vibrational patterns can involve multiple types of motion.

References

1. Pedregosa, F. *et al.* Scikit-learn: Machine learning in Python. *Journal of Machine Learning Research* (2011).
2. NPoM Calculator | NanoPhotonics Centre. https://www.np.phy.cam.ac.uk/npom-calculator.
3. Elliott, E. *et al.* Fingerprinting the Hidden Facets of Plasmonic Nanocavities. *ACS Photonics* **9**, 2643–2651 (2022).
4. Benz, F. *et al.* Nanooptics of molecular-shunted plasmonic nanojunctions. *Nano Lett* **15**, 669–674 (2015).
5. 4-Nitrothiophenol | CAS#:1849-36-1 | Chemsrc. https://www.chemsrc.com/en/cas/1849-36-1_25986.html.
6. 5-Amino-2-benzimidazolethiol | CAS#:2818-66-8 | Chemsrc. https://www.chemsrc.com/en/cas/2818-66-8_632857.html.
